# Medical Applications of Graph Convolutional Networks Using Electronic Health Records: A Scoping Review

**DOI:** 10.7759/cureus.117173

**Published:** 2026-09-29

**Authors:** Garrik Hoyt, Noyonica Chatterjee, Zachary N Davis, Fortunato Battaglia, Paramita Basu

**Affiliations:** 1 Computer Sciences, Lehigh University, Bethlehem, USA; 2 Catastrophe Modeling and Resilience, Lehigh University, Bethlehem, USA; 3 Medical Sciences, Hackensack Meridian School of Medicine, Nutley, USA; 4 Neurology and Medical Sciences, Hackensack Meridian School of Medicine, Nutley, USA; 5 Preclinical Sciences/Infectious Diseases, New York College of Podiatric Medicine, New York, USA; 6 Basic Biomedical Sciences, Touro College of Osteopathic Medicine, New York, USA

**Keywords:** ai application in electronic health record data, gcn in ehr, graph convolutional networks, intensive care unit data, medical ai

## Abstract

Electronic health records (EHRs) contain complex, interconnected patient data that traditional analytical methods often fail to fully leverage. Graph convolutional networks (GCNs) offer a promising machine learning approach for modeling intricate relationships within tabular, multimodal clinical data to improve medical decision-making and patient outcomes. This scoping review presents the first comprehensive synthesis of current research applying GCNs to EHR data for medical applications. We conducted a systematic literature search across medical databases (MEDLINE Ultimate) and computational academic databases (Academic Search Ultimate, Business Source Complete, Supplemental Index, and Complementary Index), using combined search terms for GCNs and EHRs. Following the Preferred Reporting Items for Systematic reviews and Meta-Analyses extension for Scoping Reviews (PRISMA-ScR) protocols, we screened 56 database-identified papers and five additional articles from citation review. After independent abstract screening and full-text review by multiple reviewers, 25 peer-reviewed English-language studies were included in the final analysis. Medical informatics and critical care were the most common application domains. Clinical tasks performed focused on diagnosis prediction, automated medical coding, mortality risk assessment, and medication recommendation. Hybrid architectures integrating GCNs with models such as Bidirectional Encoder Representations from Transformers (BERT) and recurrent neural networks were employed in 60% of studies. ICU EHR datasets were the most frequently used data source. Temporal trends revealed growing research interest, with eight publications (32% of the total corpus) appearing in early 2024. GCNs demonstrated measurable performance improvements in predictive accuracy, drug interaction identification, and risk stratification over other machine learning approaches. Enhanced clinical decision-making was supported through automated extraction of complex data relationships and improved handling of unstructured clinical text, with applications in critical care, oncology, hematology, and cardiovascular domains. Reported limitations included data-related challenges (68%), model-related issues (60%), and application-level barriers (28%), involving data heterogeneity, model generalizability, and clinical integration. GCNs effectively leverage complex relationships within EHR data to support clinical decision-making across diverse medical domains. The predominance of hybrid architectures and measurable performance improvements demonstrate the technology’s potential to transform healthcare delivery by improving diagnostic accuracy, personalizing treatment recommendations, and facilitating early identification of high-risk patients requiring proactive intervention. A phased, clinically grounded implementation strategy, supported by strong data governance, multidisciplinary integration, and hybrid AI architectures, will be essential for realizing GCNs’ full potential in real-world healthcare settings.

## Introduction and background

The US healthcare system faces systemic challenges, including inefficiencies, specialist consultation delays, and financial burdens that result in suboptimal health outcomes [1,2]. Graph neural networks (GNNs) applied to multimodal electronic health records (EHRs) offer a promising approach to address these inadequacies, with GNN derivatives having outperformed established predictive models in emergency department triage, heart failure pharmacotherapy optimization, hospitalization length-of-stay prediction, specialty consultation forecasting, and identification of hospital-acquired infection and ICU readmission risks [3-7].

GNNs perform inference on graph-structured data, in which information is represented as a network of nodes (entities such as patients, diagnoses, or medications) connected by edges that capture the relationships between them. They do so by learning representations that reflect how these entities depend on and relate to one another [8,9]. This learning process is achieved through graph convolution, the operation by which a model combines information from a node’s neighbors to update that node’s own representation. Most GCNs implement graph convolution through message passing: each node iteratively gathers information from its neighboring nodes and folds it into its own representation, so the resulting model captures both the structure of the network and the underlying features of each entity within it [9]. This spatial, neighbor-aggregation approach is one of two broad strategies used to define graph convolution; the other, described below, defines the operation mathematically through the graph’s spectral properties.

GNNs can be classified into four primary groups based on their underlying architecture: recurrent GNNs, convolutional GNNs, graph autoencoders, and spatial-temporal GNNs [9]. Foundational work by Gori et al. and Scarselli et al. introduced recursive architectures that update node representations using neighbor information until reaching stable fixed points [10,11]; more recent developments within the convolutional GNN category include graph convolutional networks (GCNs), graph attention networks (GATs), and graph recurrent networks [12-14]. Graph convolution was first formalized by Bruna et al. using a spectral-based approach, which defines the operation through graph Laplacian eigen decomposition; this method is mathematically well-grounded but computationally expensive and scales poorly to large graphs [15,16]. Defferrard et al. later extended this work using Chebyshev polynomials to approximate spectral convolutions more efficiently [15,16], and Kipf and Welling built on these advances to show that the spatial, message-passing formulation described above achieves strong performance on tasks such as semi-supervised node classification while remaining far more computationally tractable [12,16]. Spectral-based approaches define convolutions through graph Laplacian eigen decomposition but are computationally expensive [15,16], whereas spatial-based GCNs aggregate neighbor information directly through message-passing schemes, capturing both structural and feature information [9,12,17]. Because of this favorable balance between representational power and efficiency, GCNs have become the predominant graph-based architecture applied to structured clinical data such as EHRs and are the focus of this review.

EHRs digitally store demographics, medical history, medications, and laboratory results, but their heterogeneous data types present significant integration challenges for conventional machine learning approaches. Figure 1 illustrates the generalized process of applying GCNs to EHRs, demonstrating how graph construction captures relationships within patient data while convolution mechanisms extract representations that reveal meaningful connections. To the best of our knowledge, this is the first scoping review of medical applications of GCNs to EHR data that aims to provide a foundation for understanding current methodologies, data sources, and medical domains of the applications, establishing a baseline for future research as this field continues to expand.

**Figure 1:**
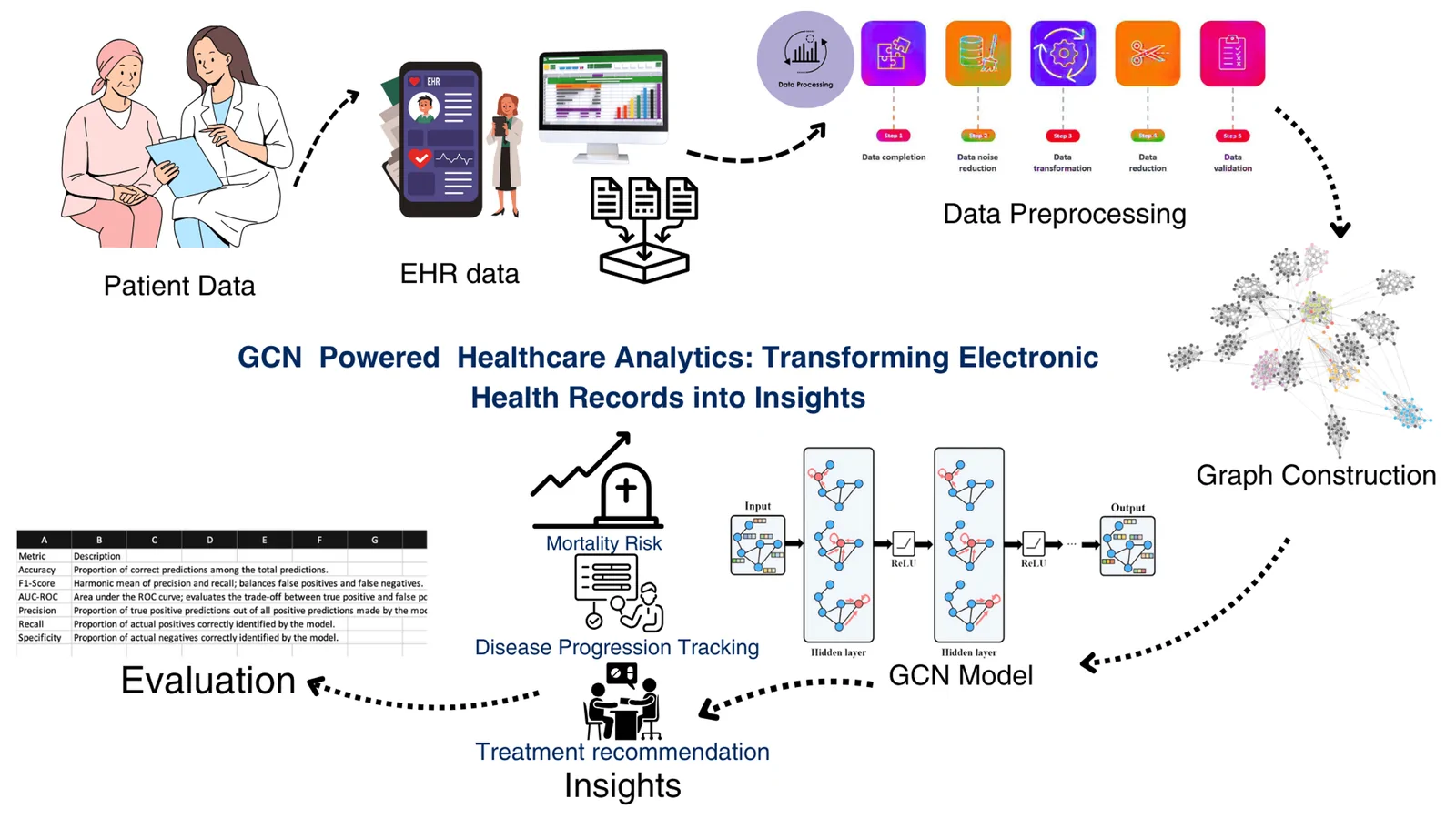
Process diagram of how GCNs are used to extract insight from patient data. First, a graph representation of the data is constructed from the EHRs. Next, a GCN performs convolution operations to derive information from the relationships between neighboring nodes in the graph. Results can be used to make informed decisions and gain insight into potential causal factors. EHR, electronic health record; GCN, graph convolutional network This figure was created using Canva (Canva Pty Ltd., Sydney, Australia).

## Review

Methods

Search Strategy 

Since this is a newly emerging field, a comprehensive scoping review of studies covering GCNs trained on EHR data in different medical fields was conducted to summarize the existing body of knowledge. The Preferred Reporting Items for Systematic Reviews and Meta-Analyses Extension for Scoping Reviews (PRISMA-ScR) protocol was used to guide the selection of studies included in the review [18]. Lehigh University’s ASA Library Catalog was queried for papers for this review. This database search was performed in April 2024 using the search strategy described in Table 1.

**Table 1:** Literature search strategy for scoping review. EHR, electronic health record; GCN, graph convolutional network

Search component	Strategy details
Target databases	MEDLINE Ultimate, Academic Search Ultimate, Business Source Complete, Supplemental Index, and Complementary Index
Search query (Boolean/phrase)	("graph convolutional" OR "GCN") AND ("Electronic health record" OR "EHR" OR "Electronic medical record" OR "EMR" OR "electronic health data")
Language filter	English language only
Publication status	Peer-reviewed papers
Date range constraints	No strict date limit applied (all retrieved results naturally fell within the past five years, up to April 2024)
Exclusion criteria	Papers with no GCN architecture, no EHR data, lack of full-text access, or determined to be out of scope (i.e., nonmedical tasks, opinion pieces, study protocols, reviews, or perspectives)
Supplemental identification	Manual citation tracking of identified review articles to capture highly relevant missed publications (categorized as “Other”)

This is a fast-moving subfield: GCN architectures and their clinical applications to EHR data are being published at an accelerating rate, with our own results showing the highest single-year article count occurring in just the first four months of 2024, as shown in the overview of study characteristics. Given this pace of change, any fixed search cutoff necessarily captures a snapshot rather than a fully current census of the literature. We selected April 2024 as our cutoff to allow adequate time for full-text screening, independent data extraction, and consensus-based resolution of discrepancies across our review team prior to submission. We therefore anticipate that additional relevant studies have been published since this date, and we recommend that readers interpret the findings below as a baseline characterization of the field as of early 2024, establishing a foundation against which future updates, whether periodic re-reviews or a living-review approach, can track the field’s continued expansion.

Additional references from a review paper were included [19-23]. These papers were categorized as “Other” since the database source was not part of the initial search. Papers from the following databases were included in the results: MEDLINE Ultimate, Academic Search Ultimate, Business Source Complete, Supplemental Index, and Complementary Index. MEDLINE Ultimate includes PubMed’s content but also adds full-text access to a wider range of biomedical journals. Academic Search Ultimate is a multidisciplinary database that includes several other EBSCO databases, such as Academic Search Complete, CINAHL Ultimate, EBSCOhost, ACM Digital Library, and Google Scholar. Business Source Complete is EBSCO’s scholarly, full-text business database that includes scholarly journals, healthcare business intelligence, and peer-reviewed research information. Complementary Index includes metadata from primary publishers for searching.

Supplemental Index includes EBSCO-enhanced metadata for journals and magazines (including peer-reviewed journals) not available in Academic Search or Business Source, as well as books, newspapers, and more.

This review focused on papers that applied GCN models to different medical domains, using EHRs as a data source. The exclusion criteria used for the review process included no GCN, no EHR data, no full-text access, and out of scope. Here, “out of scope” refers to a paper that did not focus on performing a specific medical or healthcare-centric task. Abstracts were reviewed prior to full-text assessment, during which papers were categorized according to our organizational schema.

Study Selection

The query used to retrieve papers was ("graph convolutional" OR "GCN") AND ("Electronic health record" OR "EHR" OR "Electronic medical record" OR "EMR" OR "electronic health data"). This query was limited to peer-reviewed papers written in English. The publication date range of the query was not limited, since the results were all within the past five years. Consistent with PRISMA-ScR guidance, formal critical appraisal or risk-of-bias assessment of the included studies was not performed, and accordingly, a risk-of-bias instrument was not applied to individual studies. The quality assessment of the data extraction was done informally using three reviewers as described here. After removing duplicates, one investigator (GH) reviewed all abstracts to verify the relevance of each article and excluded those that did not involve a medical application. After initial screening, the full texts of all remaining studies were examined independently by two reviewers (GH and PB); discrepancies were resolved by consensus using a third study member (FB). At this stage, articles that involved method descriptions, study protocols, reviews, perspectives, or opinion pieces were removed. Finally, the references in the review articles were searched individually to check whether important publications were missed in the database search.

The library catalog query resulted in 56 papers. Five additional papers were included after being found in a review paper [19-23]. We performed a full-text review of 25 papers after excluding 35 papers. One paper was excluded for being out of scope for this review, as it focused more on blockchain and security [24]. The inclusion and exclusion criteria used for screening and selection of the studies for this scoping review are listed in Table 2. The full process is outlined as a PRISMA flow diagram in Figure 2.

**Table 2:** Study inclusion and exclusion criteria. CNN, convolutional neural network; EHR, electronic health record; EMR, electronic medical record; GCN, graph convolutional network; LSTM, long short-term memory

Criterion type	Criterion description	Operational context/examples
Inclusion	Application of GCN models	Studies must explicitly employ GCN architectures or their immediate derivatives.
	Utilization of EHR data	EHRs, electronic medical records, or granular health data networks must serve as the primary source dataset.
	Medical/healthcare focus	The underlying computational task must directly optimize or analyze an explicit clinical domain or medical application.
	Peer-reviewed literature	Only certified peer-reviewed academic articles were eligible for full extraction.
	Language constraint	Limited strictly to studies available in full-text English.
Exclusion	No GCN architecture	Traditional deep learning or standalone network models (e.g., vanilla CNNs and independent LSTMs without a graph element) were omitted.
	No EHR/EMR source data	Studies running graph networks strictly on abstract molecular networks, raw imaging data, or text corpora without clinical history links.
	Out-of-scope tasks	Informational architectures executing platform-wide framework security, broad nonclinical data pipelines, or blockchain management.
	Missing full-text access	Abstracts or reference listings with completely restricted full-text access.
	Non-original studies	Expert opinions, systematic/narrative reviews, forward-looking perspectives, or tentative study protocols.

**Figure 2:**
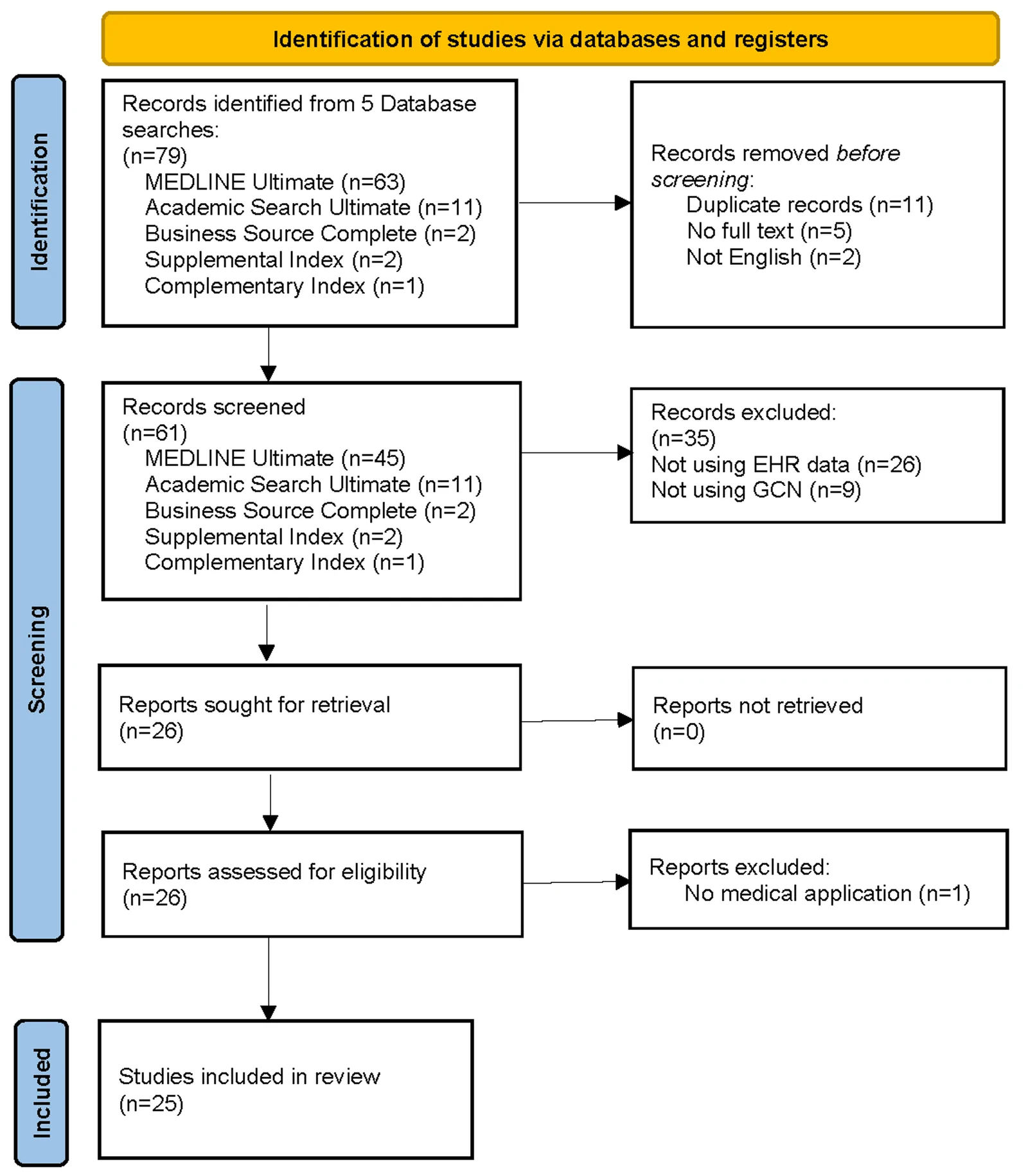
PRISMA flowchart for the literature search and selection of studies for the review. PRISMA, Preferred Reporting Items for Systematic reviews and Meta-Analyses

Data Extraction and Analysis

The PRISMA-ScR Checklist, consisting of 20 essential and two optional items to include in research reports [18], was used to ascertain that the reporting guidelines were met in order to increase methodological transparency and uptake of research findings, improving replicability and ensuring completeness.

Data extraction for all articles was done by one reviewer (GH or PB) and cross-validated by the other. Analyses were performed by three investigators (GH, NC, and ZD), two of whom were assigned randomly to each study to reduce bias. Papers were categorized by the medical area of application according to the nature of the task performed and insight provided by the proposed model. Whether studies used only a GCN in their proposed solution or a hybrid approach using multiple different model architectures was recorded. The additional models used in papers proposing a hybrid solution were categorized by model type. Data sources for each paper were documented and summarized. Review and categorization data were stored in a spreadsheet before being loaded into RStudio version 2022.12.0 to summarize and visualize the data. Descriptive summaries (frequency counts and percentages across medical domain, task type, data source, and limitation category) were generated using base R/dplyr summarization functions, and figures were produced using the ggplot2 package. Bar charts and the stacked bar chart of limitation subcategories were generated from these summarized counts. Since our analysis is descriptive only, with no meta-analysis, meta-regression, or inferential testing performed, none of the inferential statistical procedures or packages were applied, consistent with the descriptive nature of this scoping review.

Results

Overview of Study Characteristics

Analysis of the current literature shows that applying GCNs with EHR data to perform medical tasks is a recently developing trend (Figure 3). Although there are not many published papers on this subject, eight articles selected for this review were published from January through April 2024.

**Figure 3:**
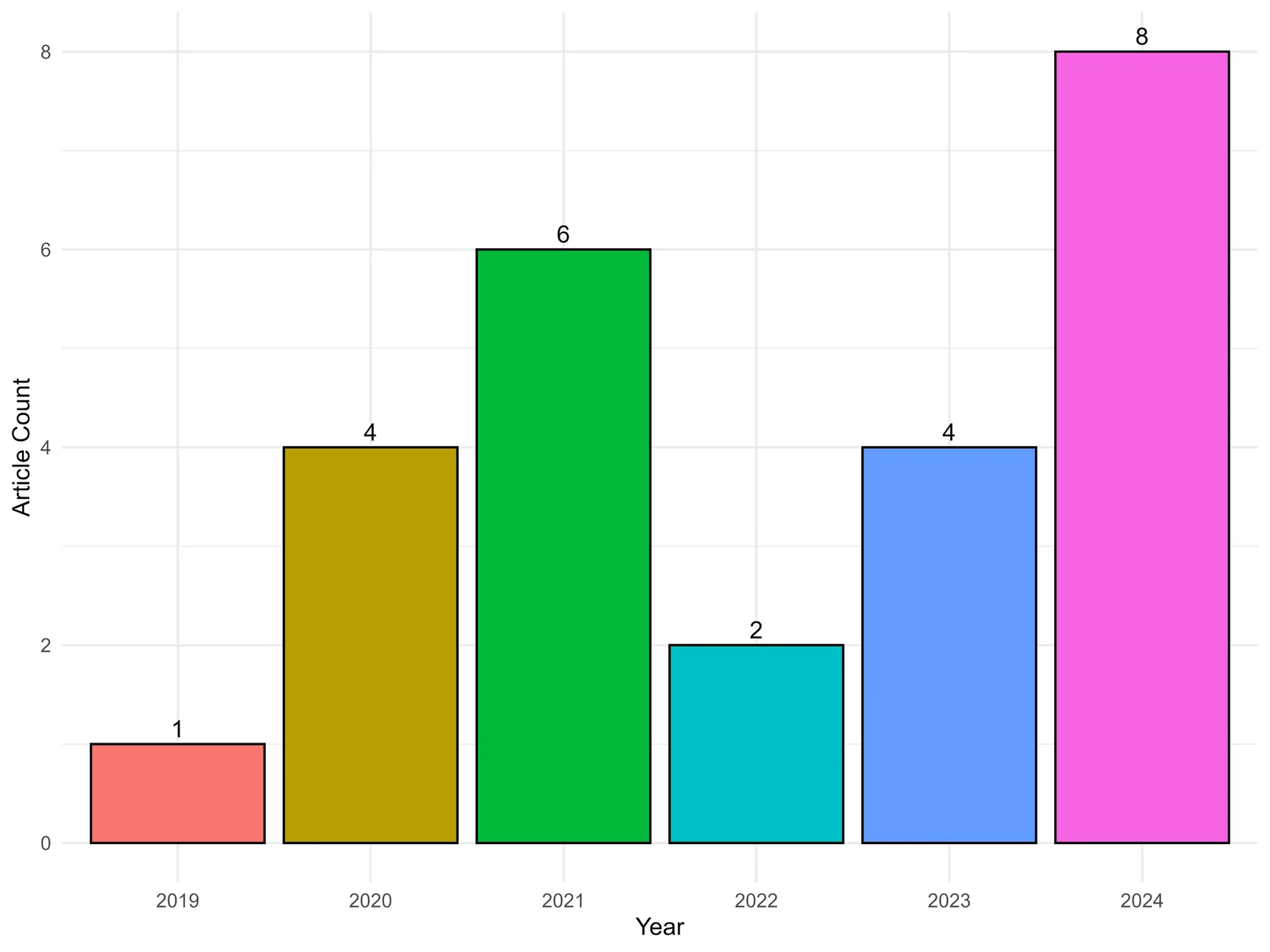
Distribution of included articles by year. The cutoff date for selection was April 27, 2024.

Hybrid Models

The various medical areas of application of GCNs with EHR data covered in the included studies were categorized based on the task performed by the model. While many of the studies utilized ICU EHR databases, articles that were not focused on a specific medical domain were categorized as general medical informatics, which broadly encompasses the use of technology to improve healthcare. Many models in this category focused on diagnosis prediction or code classification [20,21,23,25-31]. Two of these models performed text processing tasks [32,33]. Other tasks performed in this field included similar patient retrieval, medicine recommendation, terminology base enrichment, and relation classification [31,34-37]. Models applied to the area of critical care performed mortality, readmission, and decompensation prediction [37,38]. Two studies in hematology used GCNs to perform clinical risk assessment and relation classification [39,40]. The distribution of all studies focused on other domains is displayed individually in Figure 4 [41-45].

**Figure 4:**
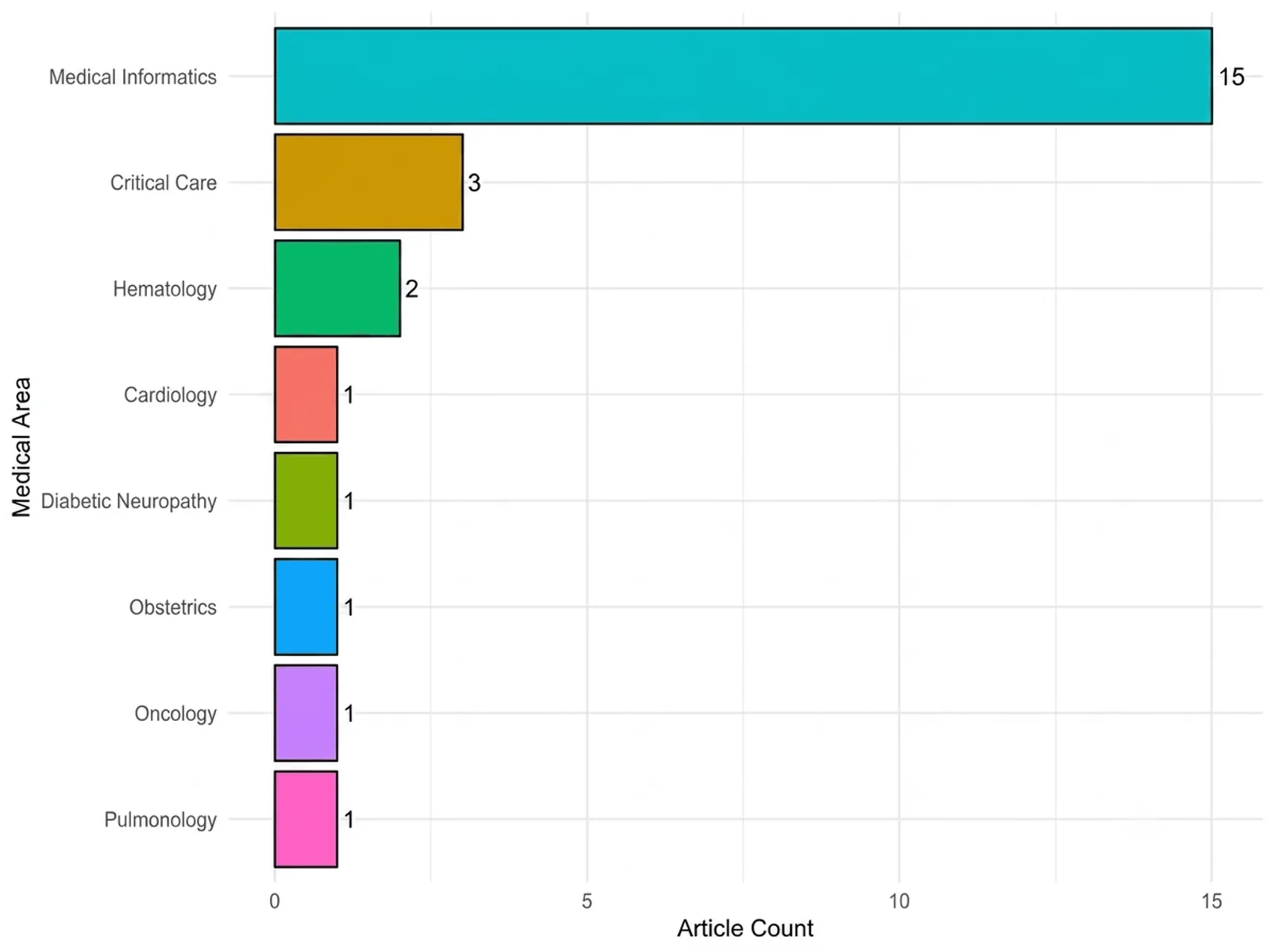
Distribution of the application of GCNs to various medical domains identified by the review. GCN, graph convolutional network

An overview of the main findings from reviewing the 25 papers is presented in Table 3, and additional details of these studies are provided in Table 4. Table 3 also shows that hybrid models are commonly applied to problems in this domain. Many studies use publicly available benchmark datasets, such as MIMIC-III and MIMIC-IV [25,26]. Fifteen of 25 papers surveyed utilized multiple machine learning models to bolster GCN performance (Table 3). Six papers used a Bidirectional Encoder Representations from Transformers (BERT) language model as part of their proposed system [35,37,42,44,46-49]. Other methods found in multiple studies include RNN-based models and LSTM models [22,29-32]. These results show that the use of multiple types of machine learning models is the most common approach within our review.

**Table 3:** Summary of GCN use across multiple studies using EHR data. BERT, Bidirectional Encoder Representations from Transformers; BiRNN, bidirectional recurrent neural network; C-EMRs, electronic medical records; CDS, clinical decision support; CNN, convolutional neural network; COEMRs, Chinese obstetric electronic medical records; COPD, chronic obstructive pulmonary disease; CRF, conditional random field; EHR, electronic health record; EMR, electronic medical record; GCN, graph convolutional network; GloVe, Global Vectors for Word Representation; ICD, International Classification of Diseases; LSTM, long short-term memory; MGAE, multi-granularity attention encoder; MIMIC, Medical Information Mart for Intensive Care; MLP, multilayer perceptron; MR, movie reviews; NGS, next-generation sequencing; NSCLC, non-small cell lung cancer; R8, Reuters-8; R52, Reuters-52; RNN, recurrent neural network; UTP, UT Physicians; 20NG, 20-Newsgroups

Reference	Dataset used	Task performed	Medical area	Data input	Clinical application of output	If hybrid, which other models?	Limitations
[21]	Real-world data	Diagnosis prediction; clinical prediction of patients’ future health conditions	General medical informatics	Patient health records from healthcare facilities; discharge summaries and progress notes (medical problems/pathology, diagnostic tests, treatments)	Leverage complex relationships within patient data (medical history, lab results, imaging). Accurate prediction of future health conditions and diagnoses. Consideration of temporal progression of diseases. Interdependence of different medical factors. More precise and proactive patient care compared to traditional methods.	Standalone	Data-related (imbalance)
[22]	MIMIC-III	Automated diagnosis	General medical informatics	Health-related data from ICU patients; discharge summaries and progress notes (medical problems/pathology, diagnostic tests, treatments)	Comprehensive and nuanced understanding of a patient’s health status. Capturing complex relationships between diagnoses, medications, and other clinical data points within the EHR. Improved diagnostic accuracy. Personalized treatment planning. Better prediction of future health outcomes.	Standalone	Model-related + data-related + application-related
[23]	MIMIC-III	Decompensation prediction; analysis of complex relationships within medical data over time	Critical care	Health-related data from ICU patients; discharge summaries and progress notes (medical problems/pathology, diagnostic tests, treatments)	Accurate prediction of potential complications in chronic disease patients. Proactive interventions based on predictions. Identification of high-risk patients for prioritizing care and monitoring. Guided treatment decisions based on predicted risk of decompensation. Timely interventions leading to improved patient outcomes.	RNN	Application-related + data-related
[24]	Real-world data; MIMIC-III	Diagnosis	General medical informatics	Patient health records from healthcare facilities; health-related data from ICU patients; discharge summaries and progress notes (medical problems/pathology, diagnostic tests, treatments)	Effective capture of complex relationships between different medical data points. Holistic consideration of patient records rather than isolated symptoms. More accurate diagnoses. Improved decision-making for healthcare providers.	Mutual Attentive Network	Model-related + data-related
[27]	MIMIC-III	ICD classification	General medical informatics	Health-related data from ICU patients; discharge summaries and progress notes (medical problems/pathology, diagnostic tests, treatments)	Accurate capture of the hierarchical structure of medical diagnoses within the ICD code system. Precise and reliable automated coding of patient medical records. Improved clinical decision-making. Enhanced research analysis. Better healthcare quality through accurate diagnosis categorization and data analysis.	CNN	Model-related + data-related
[28]	CHECK	Diagnosis; self-diagnosis service based on EHRs for online users	General medical informatics	Real-world EHR data built from electronic clinical notes collected from several hospitals offline, including chief complaints, clinical notes, and diagnosis results written by clinicians	Effective capture of complex relationships between various clinical data points. Inclusion of symptoms, medications, and patient demographics. More accurate and comprehensive self-diagnoses for online users. Earlier detection of diseases. Improved treatment planning. Better patient engagement. Analysis of a wider range of medical information beyond just symptoms. Accessible and user-friendly.	Standalone	Model-related + data-related
[29]	MIMIC-III	Mortality prediction	Critical care	Health-related data from ICU patients; discharge summaries and progress notes (medical problems/pathology, diagnostic tests, treatments)	Early identification of high-risk patients. Timely interventions. More informed treatment decisions by healthcare providers. Improved patient outcomes. Proactive management of critical cases. Facilitation of end-of-life care discussions.	Standalone	Model-related
[30]	MIMIC-III; MIMIC-IV	Diagnosis prediction	General medical informatics	Health-related data from ICU patients; discharge summaries and progress notes (medical problems/pathology, diagnostic tests, treatments)	Integration of medical knowledge and patient data. Generate highly accurate diagnoses, especially for complex conditions. Incorporate intricate relationships between symptoms, diseases, and medical concepts. Improved decision-making. Potentially earlier intervention. Support clinical decision-making. Personalized treatments.	RNN	Model-related
[31]	MIMIC-III; UTP	Diagnostic code clustering; diagnostic code prediction; visit severity classification	General medical informatics	Health-related data from ICU patients; discharge summaries and progress notes (medical problems/pathology, diagnostic tests, treatments); clinical notes from UTP	Extraction of complex, meaningful patterns from EHRs. Consideration of temporal relationships between diagnoses. Improved insights into patient health trajectories. Better understanding of potential disease progression. Enhanced clinical decision-making compared to traditional methods.	LSTM	Model-related + data-related
[32]	2010 i2b2/VA	Relation classification	General medical informatics	Health-related data from ICU patients; discharge summaries and progress notes (medical problems/pathology, diagnostic tests, treatments)	Automated extraction of complex relationships between medical concepts. Unstructured clinical text processed without manual feature engineering. Improved efficiency and accuracy in clinical decision-making. Deeper understanding of patient data.	LSTM	Data-related
[33]	20NG, R8, R52, OHSUMED, MR, MedLit	Text classification	General medical informatics	20-Newsgroups (20NG), R8 and R52 in Reuters; Ohsumed Corpus from MEDLINE database, which contains bibliographies of medical literature; and a short-text dataset of MR	Accurate and nuanced analysis of complex patient data. Leveraging relationships between different medical concepts within the EHR. Improved diagnosis. Enhanced treatment planning. Better risk prediction. Effective management of intricate medical conditions with multiple interacting factors.	GloVe, BiRNN	Model-related
[34]	Real-world data; CCKS-2019	Named entity recognition; identifying medical-related entities in Chinese electronic medical records	General medical informatics	Patient health records from healthcare facilities; health-related data from ICU patients; discharge summaries and progress notes (medical problems/pathology, diagnostic tests, treatments)	Leveraging unlabeled or Chinese-labeled data to generate high-quality labeled data. Improved recall rate and accuracy in identifying medical-related entities in Chinese electronic medical records. Enhanced patient care.	BERT, CRF	Model-related + data-related
[35]	Real-world data	Medicine package recommendation	General medical informatics	Patient health records from healthcare facilities; discharge summaries and progress notes (medical problems/pathology, diagnostic tests, treatments)	Quick and accurate identification of the most appropriate combination of medications. Consideration of individual medical profiles. Complex interactions between drugs. Minimization of potential side effects. Improved patient outcomes. More informed clinical decision-making.	Standalone	Model-related + application-related
[36]	NMEDW; MIMIC-III	Medication recommendation and lab test imputation	General medical informatics	Health-related data from ICU patients; discharge summaries and progress notes (medical problems/pathology, diagnostic tests, treatments)	Leverage complex relationships within patient medical data. More accurate and personalized recommendations for medication prescriptions and lab tests. Improved patient care. Optimized treatment plans. Minimized unnecessary testing.	Standalone	Application-related
[37]	eICU-CRD	ICU mortality prediction; in-hospital readmission prediction	Critical care	Health-related data from ICU patients; discharge summaries and progress notes (medical problems/pathology, diagnostic tests, treatments)	Early identification of high-risk patients. Proactive intervention. Improved patient outcomes. Potential reduction in mortality rates. Minimized unnecessary readmissions. Optimized resource allocation within the ICU setting.	BERT	Data-related (heterogeneity)
[38]	IgA Nephropathy Dataset; MIMIC-III	Similar patient retrieval for downstream CDS (diagnostic reference, treatment planning)	General medical informatics	Health-related data from IgA nephropathy kidney disease patients; discharge summaries and progress notes (medical problems/pathology, diagnostic tests, treatments)	Efficient identification of patients with similar medical profiles from large datasets. Quick access to relevant medical history, treatment plans, and outcomes. Informed decision-making for individual patients. Crucial for complex or rare conditions where finding comparable cases is essential.	MLP	Data-related (heterogeneity)
[39]	MIMIC-IV + Mayo Clinic data	Clinical risk assessment; prediction of risk of blood transfusion among hospitalized patients	Hematology	Patient health records from Mayo Clinic; health-related data from ICU patients; discharge summaries and progress notes (medical problems/pathology, diagnostic tests, treatments)	Proactive identification of patients at high risk of requiring a blood transfusion. Timely intervention. Optimized blood product allocation. Potentially improved patient outcomes. Proactive management strategies. Leveraging complex relationships within patient data over time.	Standalone	Model-related
[40]	Real-world data	Bleeding event relation classification	Hematology	Patient health records from healthcare facilities; discharge summaries and progress notes from patients with cardiovascular diseases on anticoagulant treatment (for bleeding events and their attributes)	Systematic assessment of bleeding risk. Optimized patient care and safety. Rapid and accurate identification of relationships between bleeding events and other medical factors. Better risk assessment. Improved treatment decision-making. Enhanced patient safety, especially for patients on anticoagulants.	Standalone	Model-related + data-related
[41]	Real-world data	Disease classification; disease type (syndrome) prediction based on multi-relational knowledge reasoning	Pulmonology	Patient health records from healthcare facilities; discharge summaries and progress notes (medical problems/pathology, diagnostic tests, treatments)	Leverage complex relationships between patient symptoms. More accurate and nuanced prediction of COPD disease type. Potential for earlier diagnosis. More targeted treatment plans. Effective management of overlapping symptoms with other respiratory conditions.	Standalone	Data-related (heterogeneity)
[42]	MIMIC-III	Predicting readmission for heart failure	Cardiology	Health-related data from ICU patients; discharge summaries and progress notes (medical problems/pathology, diagnostic tests, treatments)	Early identification of high-risk patients. Timely interventions. Preventive measures. Reduced likelihood of unnecessary hospital readmissions. Improved overall patient outcomes.	BERT	Data-related (imbalance)
[43]	Real-world data; SemMedDB; UpToDate; Churchill’s Pocketbook of Differential Diagnosis	Diagnostic decision support	Endocrinology - diabetic nephropathy	Patient health records from healthcare facilities; discharge summaries and progress notes (medical problems/pathology, diagnostic tests, treatments)	Improved diagnostic accuracy. Personalized treatment plans. Proactive risk stratification. CDS for identifying early signs of diabetic nephropathy. Timely intervention and disease management.	Standalone	Data-related + application-related
[44]	COEMRs; C-EMRs	Multi-label classification for diagnosis assistance	Obstetrics	Chinese Obstetric Electronic Medical Records and open dataset Electronic Medical Records	Improved diagnostic accuracy and efficiency in identifying complex, multifaceted diagnoses. Leveraging rich relationships within patient data. Better clinical decision-making and patient care. Particularly beneficial for patients with overlapping symptoms or conditions from different medical domains.	BERT	Data-related (imbalance)
[45]	Flatiron Health and Foundation Medicine NSCLC clinico-genomic database	Patient clustering; prevalence of a mutation within a given cancer type or across all cancer types	Oncology	Non-small cell lung cancer patient health records from Flatiron Health and Foundation Medicine; molecular biomarker/genomic data from NGS panel	Precise stratification of patients based on unique molecular profiles. Identification of individuals most likely to benefit from immunotherapy. Consideration of clinical factors and genetic mutations. Better treatment decision-making. Potentially improved patient outcomes.	MGAE	Model-related + application-related
[46]	MedSTS	Semantic similarity	General medical informatics	Clinical text and sentences from clinical corpus at Mayo Clinic	Accurate interpretation of complex medical language. Leveraging structured relationships between medical concepts. Improved clinical decision-making by identifying subtle differences in patient records. Enhanced reliability of diagnoses and treatment plans.	BERT	Model-related + data-related
[47]	Shanghai Hospital Development Center Clinical Indicator Terminology Base	Terminology base enrichment	General medical informatics	Patient health records from Shanghai hospital	Immediate and enhanced CDS. Accurate analysis of patient information data. Provision of evidence-based suggestions to medical practitioners.	BERT	Model-related + application-related

**Table 4:** Limitations, target audience, and proposed future work of included studies. COPD, chronic obstructive pulmonary disease; EMR, electronic medical record; EHR, electronic health record; GNN, graph neural network; ICD, International Classification of Diseases; ICU, intensive care unit; ML, machine learning; MIMIC-III, Medical Information Mart for Intensive Care III; MIMIC-II, Medical Information Mart for Intensive Care II; NLP, natural language processing; NSCLC, non-small cell lung cancer; NER, named entity recognition; UMLS, Unified Medical Language System

Paper title	Reference	Year	Limitation type(s)	Limitation details	Target audience	Future work proposed
Knowledge guided diagnosis prediction via graph spatial-temporal network	[20]	2024	Data-related; imbalance	Imbalanced labels, code hierarchy distortion, noisy text	AI researchers, healthcare providers	Rare label handling, external knowledge integration, broader applications
Multi-relational EHR representation learning with infusing information of diagnosis and medication	[21]	2021	Model-related + data-related + application-related	Challenges in handling inconsistencies between medical ontology knowledge and real-world observations, and difficulty in discovering relations among diverse medical concepts.	Clinical data scientists, healthcare informaticians, and AI researchers	Extending the method to more clinical tasks and improving relational understanding of diverse medical data.
TAGNet: temporal aware graph convolution network for clinical information extraction	[22]	2020	Data-related + application-related	Limited structural-temporal integration, dataset reliance, EMR variability challenges.	Healthcare AI researchers and clinicians.	Broader clinical use, enhanced generalization, contextual data integration.
The graph-based mutual attentive network for automatic diagnosis	[23]	2020	Model-related + data-related	Limited reliable corpora and sparse features, inadequate modeling of complex structural relations.	Chinese EMR (restricted), MIMIC-III-50 (public)	Not mentioned
Hyperbolic graph convolutional neural network with contrastive learning for automated ICD coding	[27]	2020	Model-related + data-related	Rare label performance, limited datasets tested (MIMIC-III, MIMIC-II)	NLP researchers, clinical coders, healthcare AI professionals	Test on rare-label datasets, extend beyond ICD coding tasks
Online disease diagnosis with inductive heterogeneous graph convolutional networks	[28]	2021	Model-related + data-related	Challenges with cold-start users, ambiguous symptom descriptions, long-tailed disease distribution, reliance on annotated datasets, and computational complexity.	Healthcare data scientists, ML researchers, and medical informatics professionals.	Improve symptom representation, integrate external knowledge bases, expand datasets, enhance retrieval accuracy, and deploy real-world applications.
Knowledge-aware representation learning for diagnosis prediction	[29]	2023	Model-related	Existing models struggle with long-term EHR sequence modeling and fail to capture complex visit-to-visit relationships and contextual knowledge.	Clinical researchers, data scientists in healthcare, and AI practitioners developing diagnosis prediction systems.	Explore multimodal EHR data and integrate more advanced medical knowledge graphs for enhanced diagnosis prediction.
Harmonized representation learning on dynamic EHR graphs	[30]	2020	Model-related + data-related	Sparse data, noisy UMLS mappings, and challenges with long relation lengths reduced accuracy.	Clinical NLP researchers and EHR tool developers.	Improve domain knowledge integration, joint NER-relation pipelines, and distant supervision.
Classifying relations in clinical narratives using segment graph convolutional and recurrent neural networks (Seg-GCRNs)	[31]	2019	Data-related	Relies on single-sentence data, limited generalizability with small datasets, and sensitivity to sample size.	Researchers in natural language processing and clinical informatics.	Explore multi-sentence data, use larger datasets for parameter tuning, and integrate knowledge-based features for enhanced classification.
EHR-HGCN: an enhanced hybrid approach for text classification using heterogeneous graph convolutional networks in electronic health records	[32]	2024	Model-related	High computational cost, transformer length restrictions	Biomedical researchers, NLP professionals	Improve scalability, explore additional benchmarks
A weakly supervised method for named entity recognition of Chinese electronic medical records	[33]	2023	Model-related + data-related	Challenges in noise suppression, dependency on weakly labeled data, limited external validation.	Healthcare NLP researchers, clinicians using EMR systems.	Enhance noise reduction methods, apply framework to diverse languages and datasets, explore integration with real-time EMR systems.
Graph-guided deep hashing networks for similar patient retrieval	[34]	2024	Data-related; heterogeneity	High dimensionality, EHR heterogeneity	Healthcare AI researchers, clinicians	Generalize to larger datasets, enhance hashing efficiency
Medicine package recommendation via dual-level interaction aware heterogeneous graph	[35]	2024	Model-related + application-related	Overlooked interactions between medicine packages, limited commonsense knowledge	Medical AI researchers, clinicians, healthcare data scientists	Enhance commonsense knowledge integration, expand to broader medical tasks
MedGCN: medication recommendation and lab test imputation via graph convolutional networks	[36]	2022	Application-related	Limited to specific medical entities and relations; ignores diagnosis codes for simplification.	Medical data researchers and healthcare practitioners.	Integration of diagnosis codes, inclusion of more medical entities, and consideration of temporal factors in MedGraph.
A cross-modal clinical prediction system for intensive care unit patient outcome	[37]	2024	Data-related; heterogeneity	Data heterogeneity, quality issues	Healthcare professionals, researchers	Generalizability, scalability, new modalities
Patient electronic health record as temporal graphs for health monitoring	[38]	2023	Model-related	ICU focus, limited generalizability.	Healthcare and ML researchers.	Expand to other tasks/populations.
Prediction of transfusion among in-patient population using temporal pattern based clinical similarity graphs	[39]	2024	Model-related	Limited generalizability, sparse external validation	Clinicians, AI researchers in healthcare	Broaden population, integrate multimodal data
Relation classification for bleeding events from electronic health records using deep learning systems: an empirical study	[40]	2021	Model-related + data-related	Challenges with long relation spans, sparse semantic graphs, limited annotated data, and potential model overfitting.	Clinical NLP researchers, medical informatics professionals.	Richer domain knowledge, joint NER-relation models, advanced data augmentation.
Feature aggregation-based multi-relational knowledge reasoning for COPD intelligent diagnosis	[41]	2024	Data-related; heterogeneity	Symptom complexity, data heterogeneity	Clinicians, AI researchers in healthcare	Generalize to broader diseases, enhance scalability
HR-BGCN: predicting readmission for heart failure from electronic health records	[42]	2024	Data-related; imbalance	Data imbalance, noisy and lengthy clinical notes	Cardiologists, clinical researchers, AI in healthcare professionals	Improve interpretability, extend to other diseases
Causal knowledge graph construction and evaluation for clinical decision support of diabetic nephropathy	[43]	2023	Data-related + application-related	Noise in knowledge bases, single data source dependency, lack of real-world evaluation.	Clinical decision support system developers, healthcare data scientists, nephrologists.	Temporal data modeling, integration of anatomical knowledge, refined causal weight learning.
Graph-based structural knowledge-aware network for diagnosis assistant	[44]	2022	Data-related; imbalance	Handling data imbalance, noise, and incomplete knowledge integration.	AI researchers, medical professionals, and informatics specialists.	Enhance graph models, rare disease classification, and broader knowledge integration.
DeePaN: deep patient graph convolutional network integrating clinico-genomic evidence to stratify lung cancers for immunotherapy	[45]	2021	Model-related + application-related	Challenges in interpretability of graph-based models and handling of missing EHR data effectively.	Oncology researchers and clinicians focusing on NSCLC immunotherapy.	Inclusion of socioeconomic data, development of interpretable graph AI models, and validation through pragmatic clinical trials
Incorporating domain knowledge into language models by using graph convolutional networks for assessing semantic textual similarity: model development and performance comparison	[46]	2021	Model-related + data-related	Small dataset size, challenges in handling subjective semantic similarity, limited data augmentation methods.	Clinical NLP researchers, healthcare professionals, and data scientists.	Enhancing data augmentation techniques, improving dataset annotation consistency, and exploring advanced GNNs.
From electronic health records to terminology base: a novel knowledge base enrichment approach	[47]	2021	Model-related + application-related	Dependency on external terminology bases, challenges in handling sparse or ambiguous clinical terms.	Researchers and practitioners in clinical informatics and terminology management.	Enhancing alignment algorithms, expanding terminology base coverage, and integrating real-time updates for clinical terms.

Data Sources

The reviewed studies predominantly utilized EHR databases as their primary data sources, with ICU EHR databases appearing in 14 instances and other health-related non-ICU EHR databases utilized in 13 instances across the literature. Non-EHR data sources were incorporated in five instances to complement the EHR sources. Several studies employed multiple data source categories, indicating that researchers frequently combine different types of healthcare data to enhance model performance (Table 1). The references for the studies using various types of data sources included in this analysis are listed in Table 5. The prominence of ICU databases, particularly established benchmark datasets such as MIMIC-III and MIMIC-IV, reflects both their accessibility for research purposes and the critical care setting’s rich, structured data environment that supports complex graph-based analyses.

**Table 5:** Summary of datasets used in the reviewed articles. The ICU EHR databases category includes 14 references representing 13 unique studies; reference [29] is counted twice because the study used both the MIMIC-III and MIMIC-IV databases. EHR, electronic health record; MIMIC-III, Medical Information Mart for Intensive Care III; MIMIC-IV, Medical Information Mart for Intensive Care IV

Data resources	References	Total
ICU EHR databases	[21-23,27,29-31,34,36-39,42]	14
Other health-related (non-ICU) EHR databases	[21,23,25,31,36,41-43,45-47,49,50]	13
Non-EHR data	[33-35,44,47]	5

The reviewed studies show that GCNs can effectively leverage diverse EHR data sources to address clinical challenges across multiple medical domains. The most utilized data inputs include ICU patient records, discharge summaries, progress notes, diagnostic test results, medication histories, and laboratory values. These heterogeneous data sources, when represented as graph structures, enable GCNs to capture complex relationships between patients, diagnoses, medications, and temporal health trajectories that traditional machine learning approaches may not identify as effectively.

Clinical Applications

GCN models demonstrate effectiveness across drug interaction analysis, diagnosis prediction, automated medical coding, mortality prediction, and medication recommendation systems. Their clinical utility extends beyond predictive accuracy to healthcare delivery improvements, including enhanced decision-making through automated extraction of complex relationships within multiple medical data sources and more personalized treatment recommendations. Critical care applications show promise for early identification of high-risk patients, enabling timely interventions that improve patient outcomes and optimize resource allocation (Table 3).

Specialized domains demonstrate domain-specific advantages. Oncology applications improve patient stratification based on molecular profiles for immunotherapy selection, while hematology applications enhance bleeding risk assessment for patients receiving anticoagulant therapy. Cardiovascular applications focus on readmission prediction for patients with heart failure requiring preventive interventions.

As documented in Table 3, GCN integration with EHR data demonstrates measurable improvements across diverse clinical tasks, from automated diagnosis and risk stratification to medication optimization and patient similarity matching. These applications illustrate GCN’s potential to enhance clinical decision-making by providing clinicians with more accurate and timely insights derived from complex patient data relationships. Hybrid approaches often perform better due to their ability to implement specialized models to perform specific tasks within a prediction pipeline. Target audiences and proposed future work are listed in Table 4.

Common Limitations 

The reviewed articles were categorized according to the limitations identified by the authors, illuminating persistent methodological and translational challenges in the field, as illustrated in Figure 5. Our literature analysis revealed that nearly all studies highlight recurring issues, primarily concerning data, modeling, and application domains. Articles classified as application-related predominantly addressed implementation or clinical utilization challenges. Data-related limitations were further classified into subcategories of data heterogeneity and imbalance (when explicitly mentioned). Model-related limitations encompassed issues of generalizability and reliability. It is noteworthy that data- and model-related limitations were often difficult to distinguish, resulting in dual categorization in such instances. Data-related limitations emerged as the most prevalent concern (17 instances), comprising heterogeneity issues (3), imbalance problems (3), and other data-specific challenges (11). Model-related limitations constituted the second most significant category, with 15 citations. Application-related limitations were documented less frequently, accounting for seven instances across the reviewed literature. The details of the limitations mentioned in each study included in this review are listed in Table 4. All reported percentages use the full set of included studies (N = 25) as the denominator. In addition, the limitation categories (data-related, model-related, and application-related) are not mutually exclusive; several studies were dual-categorized, which is why the percentages sum to more than 100%.

**Figure 5:**
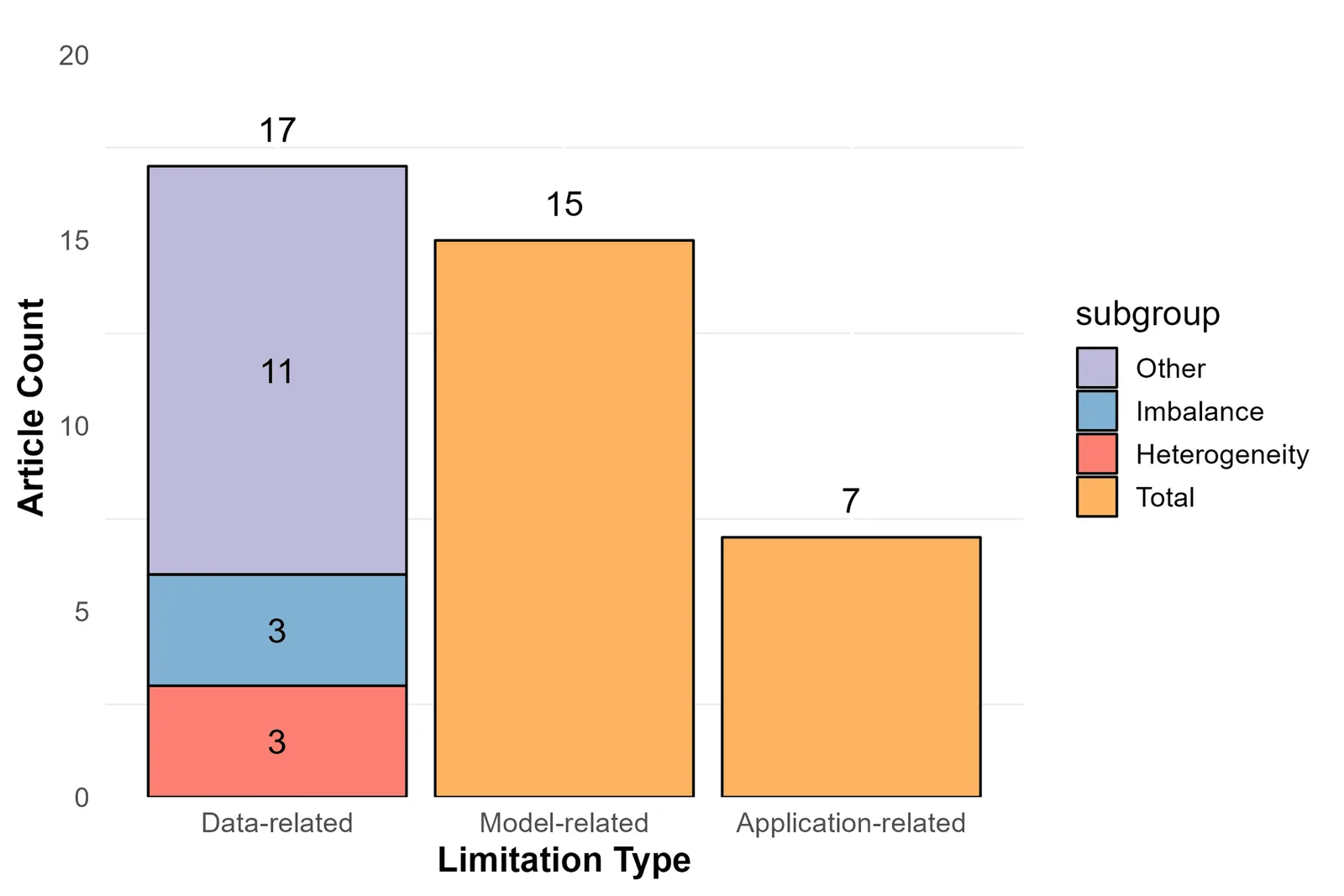
Categories of limitations identified in medical applications of GCNs in studies using EHRs. EHR, electronic health record; GCN, graph convolutional network

Discussion

This review of 25 studies demonstrates that GCNs effectively represent complex relationships in EHR data and extract meaningful insights for medical decision-making. In doing so, they support medical professionals and improve healthcare quality across diverse clinical contexts.

Principal Findings

Different medical domains covered in the included studies present implementation opportunities, with medical informatics representing the most prevalent application area, followed by critical care. Clinical applications span from disease prediction and patient stratification to therapeutic discovery and automated medical coding, and across these applications, GCNs consistently seem to outperform traditional machine learning approaches in capturing the inherent graph structure of medical data. However, it is also important to note that performance metrics were not systematically reported in all the included articles and that formal comparative analysis was not possible to perform in this report.

The field has attracted significant research interest, particularly in 2024. Hybrid approaches predominate, as researchers combine multiple machine learning models, especially BERT language models and RNN-based architectures, to enhance GCN performance. Data source analysis reveals that ICU EHR databases serve as the primary research foundation, though broad applicability across healthcare settings is also demonstrated through the use of various health-related databases, indicating the versatile potential of graph-based approaches in healthcare informatics.

Clinical Applications

EHR data inherently form multilayer graphs. Nodes represent patients, diagnoses (ICD codes), medications (RxNorm), lab tests (LOINC), and providers, connected through temporal treatment sequences, comorbidity relationships, and care pathways. Built on this structure, clinical implementations of GCNs show improvements in predicting hospital readmissions and identifying drug-drug interactions compared to conventional approaches. Temporal GATs, for example, address EHR dynamics by incorporating time-aware edge weights that decay based on medication interval durations, a feature that is critical for modeling chronic disease progression.

Several studies illustrate the scale of these gains. In a pancreatic cancer cohort, graph representations capturing chemotherapy-radiotherapy temporal edges improved survival prediction accuracy by 14% compared to sequential models [48,49]. GCNs employing spectral convolution filters, which propagate node features through graph edges while preserving topological relationships, achieved 23% higher accuracy in predicting polypharmacy side effects [50]. The MedGCN framework can automatically recommend patients’ medications based on their incomplete lab tests and can even accurately estimate lab values that have not been obtained through analysis of the complex relationships between multiple types of medical entities and their inherent features [36].

Taken together, GCNs applied to EHR data for cardiovascular disorders, tumor prognosis, and medication response show particular promise for recommending personalized therapy regimens and supporting precision prognostics [7,40,42,45,48-50]. The DeepDx GCN platform illustrates this potential at the point of care: by integrating EHR data analysis with PubMed knowledge graphs, it supports clinical decision-making through real-time diagnostic assistance, for example, by using AI-based nomograms that can improve the accuracy of predicting biochemical recurrence in prostate cancer, providing differential diagnosis suggestions ranked by evidence, and increasing efficiency and diagnostic accuracy for complex cases [51].

Deep Learning and Tabular Data

Tabular data remain challenging for deep learning architectures. Tree-based models such as XGBoost, Random Forests, and Gradient Boosting Trees remain the most accurate models for tabular data. Grinsztajn et al. identified some of the specific aspects that make tabular data difficult for deep learning models: irregular patterns, uninformative features, and non-rotationally invariant data all present significant challenges [52]. Consistent with the findings of this review, other articles have found that most deep neural network approaches for tabular data use a hybrid architecture [53]. Although tree-based models continue to prove more accurate on tabular data, deep learning architectures can be valuable as part of a specialized, hybrid solution.

Limitations

The intentionally narrow scope of this paper leaves many important aspects of how GCNs are used on EHR data unexplored. Further analyzing and categorizing the selected articles according to the convolution mechanism and type of graph representation would deliver meaningful, pragmatic information. Since we did not report performance metrics, our study is limited in its ability to perform any comparative analysis regarding model efficacy. Additionally, the small number of published papers combining GCNs with EHR data makes it difficult to deliver a comprehensive review of this subject, underscoring the need for more research in this area.

Implementation Challenges

Significant challenges persist in data standardization, model interpretability, and ethical implementation. Addressing these will require multidisciplinary collaboration to realize the full potential of graph-based AI in healthcare. Clinician resistance and missing EHR data elements disrupt graph connectivity and GCN performance, compounding operational issues [52]. The deployment of GCN-based devices adds a further layer of difficulty, since the FDA’s new AI/ML validation framework mandates rigorous testing of graph explainability methods [54].

Future Work

Deep learning models face challenges with heterogeneous, tabular EHR data. One approach converts tabular data into pixel representations for convolutional neural network training [55]. Graph convolution transformers offer a novel alternative: they use known data characteristics to guide transformers in learning hidden EHR structures [56]. Together, these approaches present opportunities to expand the knowledge base.

GCNs are also increasingly used in medical image processing for disease classification, lesion detection, and segmentation, leveraging their ability to model complex relationships within image and patient data [57]. Next-generation medical GCNs go a step further, incorporating intraoperative video graphs that track surgical instrument trajectories alongside EHR data; in early trials, this approach achieved 94% accuracy in predicting postoperative complications [57]. Separately, the NIH Bridge2AI program is developing tools and resources for processing vast amounts of medical data, including work on model susceptibility to social determinants of health-related biases [58]. Emerging solutions in federated learning, multimodal integration, and edge computing promise to overcome existing limitations, positioning graph AI as a cornerstone of next-generation evidence-based medicine.

## Conclusions

This scoping review examines the application of GCN models to EHR data across 25 studies encompassing multiple medical domains and clinical applications. Our analysis reveals that medical informatics represents the predominant application area, followed by critical care, with tasks ranging from diagnosis prediction and automated medical coding to clinical risk assessment and medication recommendation systems. The review demonstrates that hybrid approaches dominate, with most studies utilizing multiple machine learning architectures to enhance GCN performance, particularly incorporating BERT language models and recurrent neural network variants. Data source analysis confirms broad utility across healthcare settings, with studies utilizing datasets from both ICUs and non-ICU sources, and the temporal distribution of publications indicates growing research interest. The integration of GCNs with EHRs represents a paradigm shift in clinical analytics, enabling holistic analysis of patient trajectories, care networks, and biomedical knowledge, and offering healthcare organizations a transformative opportunity to leverage the complex relational structure inherent in EHR data. While current medical applications demonstrate measurable improvements in diagnosis, treatment personalization, and operational efficiency, realizing this full potential requires strategic planning that addresses data quality, model transparency, clinical integration, regulatory compliance, and equitable implementation. Organizations should start with high-impact, well-defined use cases in critical care settings while building the multidisciplinary infrastructure necessary for broader deployment. The evidence supports a measured approach that combines GCNs with proven machine learning methods within hybrid architectures, with clinicians embracing these tools while maintaining rigorous clinical oversight, best positioned to deliver transformative, data-driven care.
